# Sex Differences in Vestibular Schwannoma

**DOI:** 10.3390/cancers15174365

**Published:** 2023-09-01

**Authors:** Kathrin Machetanz, Sophie S. Wang, Linda Oberle, Marcos Tatagiba, Georgios Naros

**Affiliations:** Department of Neurosurgery and Neurotechnology, Eberhard Karls University, 72074 Tuebingen, Germany

**Keywords:** facial palsy, gender medicine, microsurgical resection, quality of life, sex, vestibular schwannoma

## Abstract

**Simple Summary:**

Vestibular schwannomas can significantly affect the quality of life (QoL) of patients pre- and postoperatively. The impact of sex on symptom- and disease-specific aspects (i.e., facial function, hearing, tinnitus, vertigo) of quality of life has not been adequately investigated. The present study analyzed the sex-specific quality of life by surveying 260 vestibular schwannoma patients pre- and postoperatively. The results demonstrated that women were significantly more affected by dizziness, headaches, anxiety, and postoperative facial palsy. However, despite the greater physical impairment, general health improved equivalently or even more in female patients than in males postoperatively. These sex differences should be considered when counseling and managing patients.

**Abstract:**

Vestibular schwannoma (VS) are equally common in men and woman. A number of epidemiological studies have reported on sex-specific aspects of incidence, tumor size, tinnitus and hearing loss. However, data on sex-specific, pre- and post-surgically quality of life (QoL) are rare. The objective of the present study was to determine sex-specific aspects on QoL in VS. Health-related QoL was analyzed in 260 patients (112 male/148 female) with unilateral sporadic VS using general (SF-36: general Short-Form Health Survey), disease-specific (PANQOL: Penn Acoustic Neuroma Quality-of-Life Scale, PANQOL) and symptom-specific (DHI: Dizziness Handicap Inventory; HHI: Hearing Handicap Inventory; THI: Tinnitus Handicap Inventory; FDI: Facial Disability Index) QoL questionnaires. Sex differences were evaluated pre- and postoperative by multi- and univariate analyses based on 200 preoperative and 88 postoperative questionnaires. Female patients were significantly more affected by dizziness, headaches, reduced energy and anxiety. Energy and balance changed similarly in both sexes after surgery. However, postoperative women tended to be more affected by facial palsy and headaches than men. Despite the greater physical impairment, general health improved equivalently or even more in female patients than in males. In conclusion, self-rated QoL in VS is significantly affected by sex and surgery. This should be taken into account when counseling VS patients regarding observation, radiotherapy, and surgery.

## 1. Introduction

Vestibular schwannomas (VS) are benign, slow-growing tumors of the vestibulocochlear nerve that—like other schwannomas—develop from Schwann cells, which are responsible for producing the protective myelin sheath around nerves. While schwannomas can occur anywhere within the body, they are most commonly localized at the head, neck, and flexor surfaces of the extremities. Although mostly non-cancerous, schwannomas may cause symptoms depending on their location including pain, numbness, tingling or weakness [1,2,3]. In this context, VS are commonly diagnosed due to a progressive loss of cranial nerve functions (i.e., hearing loss, tinnitus or dizziness) [4,5,6,7]. While historically an incidence rate of approx. 1/100,000 was assumed, more recent studies have shown changes in incidence as well as tumor size, patient age, and sex distribution at diagnosis [7,8,9]. In the past, VS were thought to primarily affect women, with larger tumors in women than in men at the time of diagnosis. Hormonal influences during menstruation and pregnancy have been discussed as a reason for this sex difference, but could not be confirmed [10,11,12,13]. Furthermore, recent studies have shown not only that VS are diagnosed at a smaller size nowadays (due to improved screening protocols for asymmetric hearing loss and better access to magnetic resonance imaging (MRI) [14]), but also that the incidence is equally distributed between men and women [8]. The fact that tumors are detected even at smaller stages has led to management becoming more patient-focused and less aggressive in recent decades. In addition to surgery, radiosurgery or a watchful waiting are now increasingly preferred [14]. The criteria for deciding among the available treatment options are typically tumor size, patient age and hearing ability [15]. Sex differences are usually not considered during decision making. However, in recent years the attention on sex-related differences in physiopathology, clinical manifestations and therapy of diseases is rising. Gender medicine does not represent a new discipline, but rather provides a new perspective on existing disciplines. In this context, previous studies demonstrated that “gender”/sex (terms are often used interchangeably) is an independent factor influencing the length of hospital stay after VS resection, with women’s average length of stay being significantly longer than that of men [16]. This may be attributed to the occurrence of vertigo and dizziness, which have been shown to affect women more than men in VS [16,17,18]. In contrast, studies inconsistently report about sex differences in the prevalence of hearing loss and tinnitus [8,19,20]. Furthermore, studies on sex-specific, patient-reported quality of life (QoL) of patients with VS are rare. However, QoL in VS can be significantly impacted due to the physical symptoms with affection of a person’s ability to communicate, work, and participate in social activities, but also by emotional and psychological distress (i.e., anxiety, depression). Due to this, the aim of this study was to investigate sex-specific QoL and related coping strategies in VS patients pre- and postoperative.

## 2. Methods

### 2.1. Demographics and Patient Characteristics

This cross-sectional study enrolled 260 patients (112 male/148 female; Table 1) with unilateral sporadic VS treated at our Neurosurgical Department between November 2019 and October 2022. Patients answered standardized questionnaires on QoL as part of a routine diagnostic. Microsurgical resection of the VS was performed in a total of 165/260 (63.5%) patients during or before the above-mentioned period using a retrosigmoid–transmeatal approach in a supine or a semi-sitting position in all cases. Patients with neurofibromatosis, previous external tumor resection or radiosurgery and incomplete questionnaires were excluded from further data analyses. The study was carried out in accordance with the recommendations of the ethics committee of the Eberhard Karls University Tuebingen (approval number 403/2021B02) and conducted in accordance with the declaration of Helsinki. 

In addition to QoL questionnaires, we recorded numerous patient- and disease-specific characteristics. Facial function was graded according to the House–Brackmann score (HB), which classifies overall facial function into ranges from 1 (normal) to 6 (total paralysis) based on the assessment of, e.g., eye closure, mouth and forehead movement [21]. Tumor side and size according to the Koos classification (1: purely intrameatal, 2: intra- and extrameatal, 3: filling the cerebellopontine cistern, 4: compressing or shifting the brainstem) were retrospectively analyzed by inspection of the MRI [22]. 

### 2.2. Health-Related Quality of Life Questionnaires

We routinely administered several quality-of-life surveys during the outpatient presentation or inpatient admission in our department, as previously described [23]: the disease-specific Penn Acoustic Neuroma Quality-of-Life Scale (PANQOL), the general Short-Form Health Survey (SF-36) as well as the symptom-specific Hearing Handicap Inventory (HHI), Tinnitus Handicap Inventory (THI), Dizziness Handicap Inventory (DHI), and Facial Disability Index (FDI).

The PANQOL consists of 26 questions, which can be answered by the patient on a Likert scale from strong agreement (5) to strong disagreement (1). Answers of the 7 domains general health (PAN-GH), pain (PAN-PAIN), anxiety (PAN-ANX), energy (PAN-ENGY), balance (PAN-BAL), facial function (PAN-FACE) and hearing (PAN-HEAR) are normalized to a scale of 0 (lowest QoL) to 100 (best possible QoL) points to determine the domain scores. Additionally, a sum score (PAN-TTL; ranging also from 0–100) can be generated from the domain scores.

The SF-36 has 36 self-assessment items estimating general QoL. The items can be categorized into the domains general health (SF36-GH), physical function (SF36-PF), social function (SF36-SF), role-physical (SF36-RP), role-emotional (SF36-RE), mental health (SF36-MH), vitality (SF36-VT) and bodily pain (SF36-BP). The answers of each domain can be converted into a score from 0–100, with a lower score corresponding to a lower QoL. Sum scores can be calculated by using the domain values, a population-based Z-score standardization and a T-score transformation. As population scores for VS do not significantly differ between the German and U.S. population, the scores of the U.S. population were used in the present study for better comparability [24].

Symptom-specific QoL was assessed by the THI, DHI and HHI. Patients have to answer 25 items in each survey ((yes (2), sometimes (1) or no (0)), which address problems due to tinnitus, dizziness and hearing function. A total score of 0–100 can be calculated, whereby a lower score indicates a better QoL. Furthermore, all patients with facial palsy were evaluated with the FDI, which contains 10 self-assessment questions. These survey items can be categorized into physical function (FDI-PF) and social function (FDI-SF). Domain scores range up to 100 points, which represents the best symptom-specific QoL.

### 2.3. Statistical Analysis

SPSS (IBM SPSS Statistics for Windows, Version 26.0. Armonk, NY: IBM Corp.) was used to perform all statistical analyses. Differences between groups (i.e., women versus men) regarding the distribution of clinical attributes such as tumor side, tumor size and patients’ age were evaluated by Chi-squared or Kruskal–Wallis tests. Multivariate analyses of covariance (MANCOVAs) were performed to evaluate the effects of SEX and pre- and postoperative groups (SURG) on QoL scores in consideration of the covariates AGE and tumor SIZE. The data were first examined for outliers and homogeneity (Levene’s test). Subsequently, a MANCOVA was performed in a two-level analysis: after testing the overall hypothesis (i.e., is there a difference between the groups?), follow-up analyses (i.e., univariate ANOVAs) were conducted to explain the group differences. Furthermore, correlation analyses were performed using Spearman’s rank correlation. Statistical significance was considered at *p* < 0.05 for each statistical test. The results are presented as mean ± SEM. 

## 3. Results

### 3.1. Patient Characteristics

A total of 260 VS patients (52.5 ± 12.5 years, 148 female) completed all QoL questionnaires (Table 1). The cohort involved similarly small (Koos 1/2: 119/260, 45.8%) and large (Koos 3/4: 146/260, 56.2%) as well as left- (137/260, 52.7%) and right-sided (123/260, 47.3%) vestibular schwannomas. In 165/260 (63.5%) patients, the VS was resected, while 95/260 (36.5%) had not undergone surgery at the time of evaluation. Tumor size correlated significantly with the decision for surgery (r = 0.62, *p* < 0.001, Spearman’s), i.e., Koos 3/4 tumors were resected more frequently than Koos 1/2 VS. There were no differences between age, tumor size or frequency of performed surgeries between male and female patients. Furthermore, the time between VS diagnosis and date of preoperative survey (time since diagnosis, TSD) and the time between surgery and date of postoperative QoL survey (time since surgery, TSS) differed not between sexes. However, the affected side differed significantly between sexes (t_(241)_ = −2.02, *p* = 0.045). While in men VS were predominantly located on the left side (67/112, 59.8%), in women the number of tumors on the right side (78/148, 52.7%) exceeded the number on the left side (70/148, 47.3%).

### 3.2. Sex Differences in General and Disease-Related QoL: SF-36 and PANQOL

**Figure 1:**
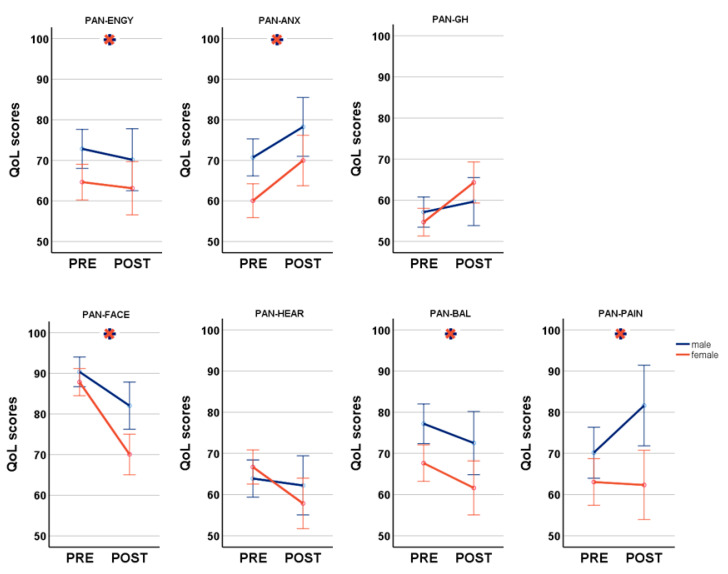
Disease-specific gender differences pre- and postoperative analyzed by the Penn Acoustic Neuroma Quality-of-Life Scale (PANQOL); PAN-ENGY: PANQOL energy; PAN-ANX: PANQOL anxiety; PANQOL-GH: PANQOL general health; PAN-FACE: PANQOL facial; PAN-HEAR: PANQOL hearing; PAN-BAL: PANQOL balance; PAN-PAIN: PANQOL pain. 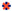 indicates significant differences between genders. Blue lines: male; orange lines: female.

Based on a total of 288 questionnaires (200 preoperative, 88 postoperative), we performed a MANCOVA to analyze the impact of SEX and SURG on PANQOL while controlling for AGE and SIZE. A significant multivariate main effect was found for SEX (F_(7,276)_ = 5.20, *p* < 0.001) and SURG (F_(7,276)_ = 8.63, *p* < 0.001). Female patients suffered significantly more from balance problems (PAN-BAL: 65.73 ± 24.5 vs. 75.85 ± 22.0; F_(1,288)_ = 11.70, *p* = 0.001) and headaches (PAN-PAIN: 62.86 ± 31.1 vs. 73.44 ± 28.0; F_(1,288)_ = 11.90, *p* = 0.001), as well as decreased energy (PAN-ENGY: 64.18 ± 24.6 vs. 72.0 ± 21.1; F_(1,288)_ = 6.53, *p* = 0.011) and anxiety (PAN-ANX: 63.24 ± 21.8 vs. 72.9 ± 23.0; F_(1,288)_ = 11.24, *p* = 0.001) in comparison to males. Furthermore, females were more affected by facial impairment (PAN-FACE: 82.12 ± 20.5 vs. 88.0 ± 16.8; F_(1,288)_ = 10.30, *p* = 0.001) although postoperative HB differed not between sexes (Table 1). Thereby, postoperative HB correlated significantly with PAN-FACE in both men (r = −0.61, *p* < 0.001; Spearman’s) and women (r = −0.75, *p* < 0.001; Spearman’s). Although the difference in hearing impairment pre- vs. postoperatively was greater in women than in men, this did not reach significance. SURG had a significant main effect on PAN-ANX (64.78 ± 23.0 vs. 73.71 ± 21.2; F_(1,288)_ = 8.22, *p* = 0.004), PAN-FACE (89.12 ± 14.3 vs. 74.75 ± 24.4; F_(1,288)_ = 28.75, *p* < 0.001) and PAN-GH (55.0 ± 17.1 vs. 64.16 ± 19.4; F_(1,288)_ = 6.22, *p* = 0.013). Notably, general health and anxiety improved postoperatively despite a facial deterioration (Figure 1). MANCOVA demonstrated a significant main effect of SEX*SURG on PANQOL (F_(7,276)_ = 2.55, *p* = 0.015) with a significant impact on PAN-FACE (F_(1,288)_ = 4.37, *p* = 0.038) with worse values postoperative and in females in follow-up ANOVAs.

**Figure 2:**
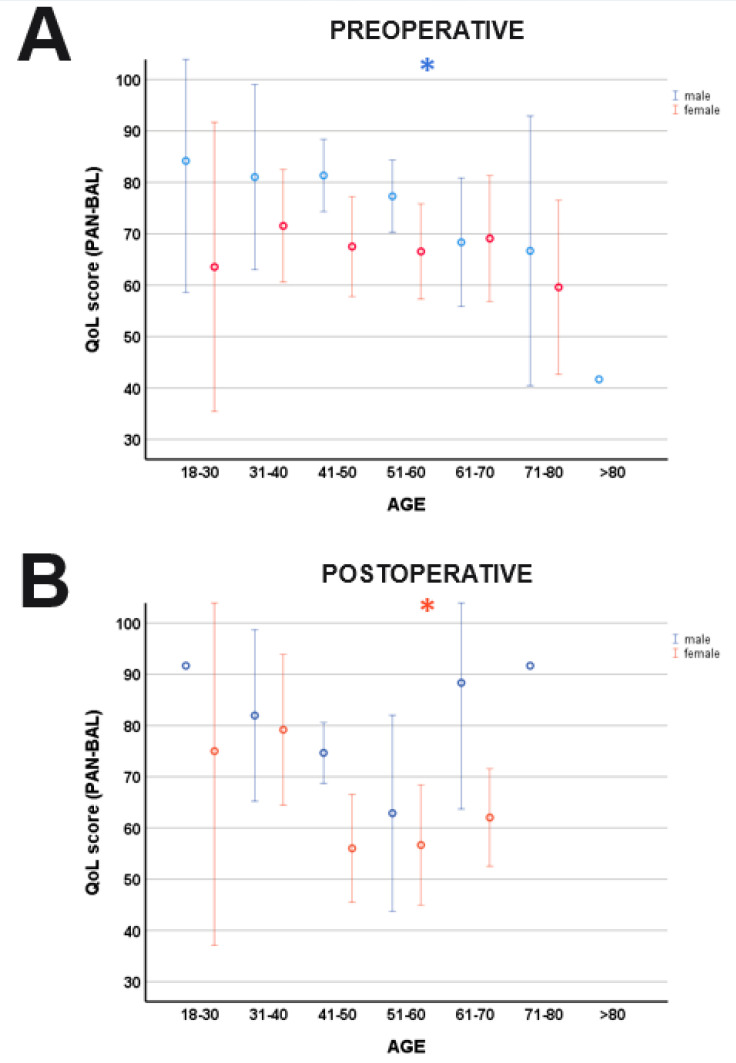
Age-related differences of balance (PAN-BAL). While there was a significant correlation between AGE and PAN-BAL in the male cohort preoperatively (**A**), postoperative women demonstrated more balance problems with increasing AGE (**B**). * indicates a significant correlation age and PAN-BAL. Blue lines: male; orange lines: female.

PANQOL was significant affected by the covariates AGE (F_(7,276)_ = 4.96, *p* < 0.001) and SIZE (F_(7,276)_ = 2.13, *p* = 0.041). QoL was more influenced by anxiety in younger patients (PAN-ANX: F_(1,288)_ = 4.67, *p* = 0.031) than in the elderly. However, separate sex-specific correlation analyses could not reveal significant correlations between AGE and PAN-ANX neither for pre-/postoperative nor female/male. In contrast, increasing AGE had a negative effect on balance (PAN-BAL: F_(1,288)_ = 8.59, *p* = 0.004), hearing (PAN-HEAR: F_(1,288)_ = 6.72, *p* = 0.010) and facial function (PAN-FACE: F_(1,288)_ = 7.40, *p* = 0.007). Post hoc, sex-specific correlation analyses detected significant correlations between AGE and PAN-BAL in the postoperative female cohort (r = −0.28, *p* = 0.043; Spearman’s) and preoperative male patients (r = −0.27, *p* = 0.010; Spearman’s) (Figure 2). PAN-GH (F_(1,288)_ = 7.03, *p* = 0.008) and PAN-ENGY (F_(1,288)_ = 4.59, *p* = 0.033) were significantly influenced by the SIZE with better values in large tumors (PAN-ENGY: r = 0.13, *p* = 0.025; PAN-GH: r = 0.23, *p* < 0.001). 

The MANCOVA applied to the SF36 subdomains with the same factors and covariates demonstrated a significant effect only for AGE (F_(7,275)_ = 3.55, *p* = 0.001). Follow-up ANOVAs confirmed a significant impact on SF36-PF (F_(1,288)_ = 10.05, *p* = 0.002) and SF36-GH (F_(1,288)_ = 4.85, *p* = 0.029), both decreasing with increasing age.

### 3.3. Symptom-Specific Sex Differences: HHI, THI, DHI and FDI

**Figure 3:**
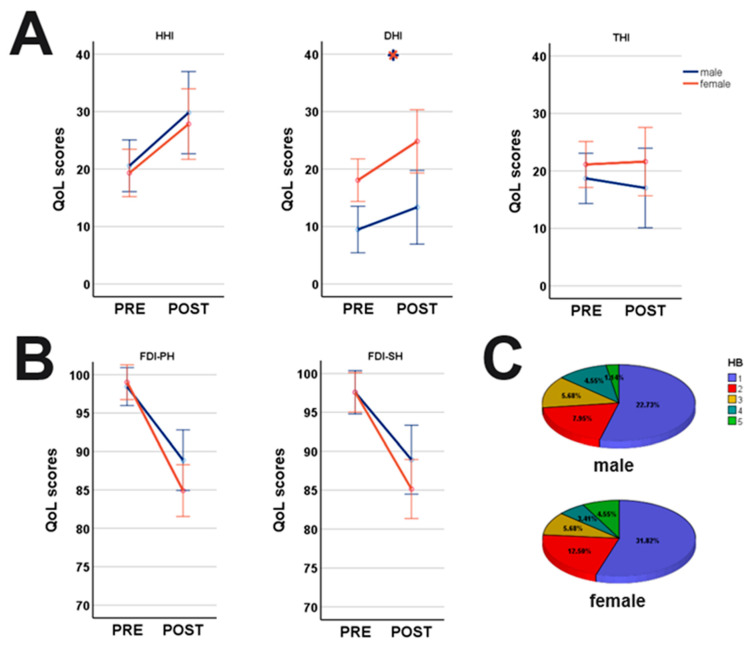
Symptom-specific gender-differences pre- and postoperative. (**A**) Women were significantly more affected by dizziness (DHI) than men, while no gender-differences were detected for HHI and THI; (**B**) facial function decreased postoperative in both genders with women more affected; (**C**) postoperative HB scores demonstrated no differences between genders (H = 0.01, *p* = 0.930; Kruskal–Wallis); HHI: Hearing Handicap Inventory, DHI: Dizziness Handicap Inventory, THI: Tinnitus Handicap Inventory; 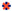 indicates significant differences between genders. Blue lines: male; orange lines: female.

A MANCOVA was performed to determine the effects of SEX and SURG on the handicap inventories (i.e., DHI, HHI and THI) (Figure 3). While SEX (F_(3,280)_ = 9.73, *p* < 0.001) demonstrated a significant impact on DHI (21.56 ± 1.7 vs. 11.84 ± 1.9; F_(1,288)_ = 16.06, *p* < 0.001) with more dizziness in female patients, SURG (F_(3,280)_ = 5.17, *p* = 0.002) affected hearing function (19.96 ± 21.6 vs. 28.50 ± 21.7; F_(1,288)_ = 8.75, *p* = 0.003) and DHI (14.34 ± 19.3 vs. 19.57 ± 21.1; F_(1,288)_ = 6.04, *p* = 0.001) with worse values postoperative. AGE (F_(3,280)_ = 9.73, *p* < 0.001) affected HHI (F_(1,288)_ = 3.93, *p* = 0.048) with a negative effect in the elderly. Tinnitus was only affected by the SIZE (F_(1,288)_ = 5.69, *p* = 0.018).

Surgery (SURG) negatively affected both the FDI subscores physical (FDI-PH; F_(1,288)_ = 51.57, *p* < 0.001) and social disability (FDI-SH; F_(1,288)_ = 32.32, *p* < 0.001) of facial palsy. However, SEX, AGE or SIZE had no effect on the FDI.

## 4. Discussion

Gender medicine does not represent a new discipline, but rather offers a new perspective on existing disciplines respecting sex differences on pathophysiology, symptoms, prevention and therapy of diseases [25,26]. Important sex differences have already been identified in cardiovascular disease, osteoporosis, and pharmacokinetic properties [25]. However, sex-specific data in vestibular schwannomas are rare. In this context, we demonstrated numerous sex differences of symptom-specific and mental QoL in VS, with women being more affected than men. Notably, despite symptom-related deterioration mainly caused by increased dizziness and facial nerve impairment, female patients reported a greater postoperative improvement in general health (PAN-GH). This improvement reached “minimal clinically important difference” (MCID) reported in the literature [27]. Hence, sex differences should be considered when counseling patients with VS offering more psychological support and dizziness/balance training especially for women.

Recent studies on vestibular diseases in general (e.g., benign paroxysmal positional vertigo, Mèniere disease, vestibular neuritis) and on VS-related vertigo in particular, have shown, consistent with our study, that women are more affected by dizziness than men [18,28]. Hormonal causes are suspected as the reason for this sex difference, since it was shown that postmenopausal women were more frequently affected by vestibular disorders. Furthermore, studies reported that patients who received hormone replacement therapy (e.g., in the context of osteoporosis) had fewer vestibular disorders [29,30]. The concept of hormone replacement therapy in postmenopausal VS patients with vertigo should be further investigated in the future—especially after previous studies could not confirm a hormonal influence on VS growth and our data demonstrated more balance problems in older women after surgery [11,13]. In this context, as a first step, a pre- and postoperative investigation of hormone levels and their fluctuations based on blood tests would be interesting to investigate the correlation with clinical symptoms (e.g., measured by questionnaires or use of an online digital diary for symptom monitoring by patients). In case of a correlation, a further step could be the substitution of hormone medications in patients with low hormone levels.

The facial deterioration rate after VS resection has been reported in the literature to be ~35%, with the tumor size as the most important predictive factor [31,32,33]. We demonstrated that female patients were significantly more affected by the manifestation of facial nerve palsy than males. The MCID, which is specified as 10 points for the PAN-FACE [27], was reached between sexes. The detected sex difference is consistent with both etiology-independent and VS-specific studies on the occurrence of facial palsy [34,35,36]. The reasons for this are possibly a higher demand on appearance in women, which leads to a greater distress and self-consciousness with anxiety and depression at the occurrence of facial palsy. Different coping strategies for women and men must also be discussed in this context. Studies often report about a predominance of problem-focused coping (i.e., try to change or eliminate a stressor) in men, while women typically use emotion-focused coping skills (i.e., try to manage emotional response to the stressor) [37,38]. Women therefore seek social support (e.g., turn to friends and relatives). Accordingly, the limited QoL in facial palsy in women compared to men may be due to the fact that women avoid social contacts according to their poor appearance and thus lose a coping strategy. However, this assumption needs to be discussed because, (i) problem-focused coping predominates in women with primary brain tumors, but also during the Gulf crisis [39,40], (ii) in patients with facial paresis of different etiologies, emotion-focused coping has been shown to improve anxiety and depression more than problem-focused coping, and (iii) despite a postoperative increase in facial impairment, our data showed an improvement of the PAN-ANX score [41]. Hence, current data related to VS-associated facial palsies are insufficient to make a conclusive assessment in this context. Further studies focusing on different coping strategies preoperatively and postoperatively in VS should be performed in the future.

Anxiety disorders are more common in women than in men [42,43]. In accordance, our data as well as further studies on brain tumor patients showed a sex difference in the occurrence of anxiety and distress, with women being more affected [44]. In line with the already described impact on vertigo, hormonal differences are considered to be a potential cause of the disparity. Thus, anxiety disorders are more common in postmenopausal than in younger women [43,45]. Animal studies suggest that a loss of estrogen is a predictor of anxiety disorders [46]. In contrast, our MANCOVA revealed higher anxiety-scores (PAN-ANX) in younger patients than in elderly patients. However, separate sex-specific correlation analyses could not reveal significant correlations between AGE and PAN-ANX. Instead, in both sexes we demonstrated a decrease in anxiety postoperatively. This improvement was more pronounced in women and is concordant with previous studies in meningiomas and benign tumors that demonstrated increased anxiety during watchful waiting [47,48]. This improvement in anxiety may also be contributory to the improvement in general health (PAN-GH), which was demonstrated despite the deterioration in symptom-specific QoL, and should consequently be taken into account when making treatment decisions.

### Limitations

The present study is limited in particular by the small number of postoperative questionnaires and the absence of a longitudinal follow-up. The sex-specific analyses refer only to observed and operated VS patients. Statements on patients treated by radiosurgery cannot be made. Furthermore, demographic aspects such as education level, socioeconomic status, ethnicity, relationship status, or treatment of psychological impairments (i.e., psychological and psychiatric co-care) were not included in the analysis.

## 5. Conclusions

Reviewing the quality of life in vestibular schwannoma patients from the perspective of gender medicine reveals sex differences in the occurrence of and coping with vertigo, facial nerve palsy, and anxiety. Attending physicians should take this into account when providing pre- and postoperative counseling and treatment to VS patients. Furthermore, additional studies should be conducted to investigate the correlation between hormone levels and symptoms as well as coping strategies in VS.

## Figures and Tables

**Table 1:** Patient characteristics.

	Total Cohort n = 260	Preoperative Quest. n = 200			Postoperative Quest. n = 88		
	112 Male/ 148 Female	Male n = 91	Female n = 109		Male n = 37	Female n = 51	
Age		52.60 +/− 12.7	53.0 +/− 12.4		50.59 +/− 11.7	50.43 +/− 11.6	
Koos 1 2 3 4	43 (16.5%) 76 (29.2%) 81 (31.2%) 60 (23.1%)	16 (17.6%) 36 (39.6%) 26 (28.6%) 13 (14.3%)	26 (23.9%) 31 (28.4%) 33 (30.3%) 19 (17.4%)	H = 0.02 *p* = 0.892	1 (2.7%) 5 (13.5%) 16 (43.2%) 15 (40.5%)	1 (2.0%) 9 (17.6%) 20 (39.2%) 21 (41.2%)	H = 0.01 *p* = 0.913
Side Left/ Right	137 (52.7%) 123 (47.3%)	56 (61.5%) 35 (38.5%)	55 (50.5%) 54 (49.5%)	X^2^ = 2.47 *p* = 0.116	18 (48.6%) 19 (51.4%)	21 (41.2%) 30 (58.8%)	X^2^ = 0.49 *p* = 0.486
Operation Yes No	165 (63.5%) 95 (36.5%)	45 (49.5%) 46 (50.5%)	50 (45.9%) 59 (54.1%)	X^2^ = 0.04 *p* = 0.847	37 (100%) 0	51 (100%) 0	X^2^ = 0 *p* = 1
TSD/TSS		1.23 +/− 2.3 y	1.16 +/− 2.2 y	H = 0.25 *p* = 0.615	1.48 +/− 2.1 y	2.37 +/− 3.1	H = 0.14 *p* = 0.137
HB I II III IV V		89 (97.8%) 2 (2.2%) 0 0 0	104 (95.4%) 5 (4.6%) 0 0 0	H = 83 *p* = 0.361	20 (54.1%) 7 (18.9%) 5 (13.5%) 4 (10.8%) 1 (2.7%)	28 (54.9%) 11 (21.6%) 5 (9.8%) 3 (5.9%) 4 (7.8%)	H = 0.01 *p* = 0.930
EOR GTR STR PR	64 (72.7%) 19 (21.6%) 5 (5.7%)	- - -	- - -		25 (67.6%) 8 (21.6%) 4 (10.8%)	39 (76.5%) 11 (21.6%) 1 (2.0%)	X^2^ = 3.19 *p* = 0.203
SF-36 physical function role physical bodily pain general health vitality social function role emotional mental health		89.78 ± 17.7 75.27 ± 36.6 76.45 ± 27.8 63.87 ± 17.5 58.52 ± 19.9 77.20 ± 25.2 73.35 ± 40.1 69.71 ± 19.3	81.06 +/− 24.2 64.45 +/− 42.3 66.58 +/− 30.8 59.73 +/− 17.6 52.16 +/− 20.5 70.64 +/− 25.8 65.14 +/− 41.7 64.62 +/− 17.9	*p* = 0.001 * p = 0.067 *p* = 0.023 * p = 0.097 *p* = 0.032 * p = 0.053 *p* = 0.089 *p* = 0.043 *	86.89 +/− 16.6 72.16 +/− 33.6 80.46 +/− 27.0 66.08 +/− 19.6 59.65 +/− 16.7 78.11 +/− 21.9 84.01 +/− 29.9 75.89 +/− 18.7	78.76 +/− 21.7 60.00 +/− 41.2 71.57 +/− 30.2 65.24 +/− 19.2 55.53 +/− 24.0 73.97 +/− 26.7 70.59 +/− 43.5 69.65 +/− 20.5	*p* = 0.079 *p* = 0.256 *p* = 0.132 *p* = 0.879 *p* = 0.425 *p* = 0.579 *p* = 0.251 *p* = 0.129
PANQOL anxiety facial general health balance hearing energy pain Total		70.54 ± 22.9 90.57 ± 14.2 56.32 ± 19.0 76.69 ± 22.0 63.46 ± 22.5 71.98 ± 21.5 70.05 ± 28.9 71.37 ± 15.6	59.98 +/− 22.1 87.92 +/− 14.3 54.0 +/− 15.3 67.05 +/− 25.7 66.17 +/− 21.0 63.80 +/− 24.2 63.07 +/− 30.2 66.10 +/− 14.6	*p* = 0.001 * p = 0.129 *p* = 0.267 *p* = 0.008 * p = 0.332 *p* = 0.015 * p = 0.086 *p* = 0.006 *	78.55 +/− 22.8 81.67 +/− 20.6 61.49 +/− 19.6 73.76 +/− 22.2 63.34 +/− 18.0 72.07 +/− 20.5 81.76 +/− 24.0 73.21 +/− 14.3	70.21 +/− 19.4 69.73 +/− 25.9 66.10 +/− 19.2 62.91 +/− 21.7 58.99 +/− 24.9 64.99 +/− 25.6 62.42 +/− 33.4 64.29 +/− 17.8	*p* = 0.027 * p = 0.027 * p = 0.420 *p* = 0.008 * p = 0.468 *p* = 0.242 *p* = 0.006 * p = 0.016 *
DHI		9.65 ± 16.3	18.26 +/− 20.8	*p* = 0.001 *	12.92 +/− 16.7	24.39 +/− 22.7	*p* = 0.011 *
THI		19.41 ± 21.1	21.71 +/− 21.5	*p* = 0.431	15.51 +/− 18.1	20.20 +/− 22.3	*p* = 0.432
HHI		20.59 ± 20.9	19.43 +/− 22.2	*p* = 0.473	29.68 +/−19.8	27.65 +/−23.1	*p* = 0.475
FDI physical function social function		98.74 ± 10.5 97.89 ± 12.1	99.27 +/− 3.3 97.87 +/− 8.1	*p* = 0.319 *p* = 0.402	88.24 +/−16.5 88.22 +/−16.3	84.31 +/− 19.6 84.47 +/− 20.4	*p* = 0.382 *p* = 0.491

* *p*-values indicate significance comparing male and female by a Chi-square (X^2^) or Kruskal–Wallis test (H). FDI: Facial Disability Index; DHI: Dizziness Handicap Inventory; HB: House-Brackmann scale; HHI: Hearing Handicap Inventory; EOR: extent of resection; GTR: gross total resection; PANQOL: Penn Acoustic Neuroma Quality-of-Life Scale; PR: partial resection; SF-36: general Short-Form Health Survey; STR: subtotal resection; THI: Tinnitus Handicap Inventory; TSD: time since diagnosis; TSS: time since surgery; FDI: Facial Disability Index.

## Data Availability

The data that support the findings of this study are available from the corresponding author upon reasonable request.

## Abbreviations

DHI: Dizziness Handicap Inventory; FDI: Facial Disability Index; GTR: gross total resection; HB: House-Brackmann scale; HHI: Hearing Handicap Inventory; MCID: minimal clinically important difference; MRI: magnetic resonance imaging; PANQOL: Penn Acoustic Neuroma Quality-of-Life Scale; PR: partial resection; QoL: quality of life; SF-36: general Short-Form Health Survey; STR: subtotal resection; THI: Tinnitus Handicap Inventory; TSD: time since diagnosis; TSS: time since surgery; VS: vestibular schwannoma.
